# Vanishing white matter disease imaged over 3 years

**DOI:** 10.4102/sajr.v23i1.1661

**Published:** 2019-02-27

**Authors:** Denny Mathew, Nasreen Mahomed

**Affiliations:** 1 Department of Radiology, University of the Witwatersrand, South Africa; 2 Department of Radiology, Rahima Moosa Mother and Child Hospital, South Africa; 3 South African Society of Paediatric Imaging (SASPI), Cresta, South Africa

## Abstract

Childhood ataxia and central nervous system hypomyelination (CACH), also known as ‘vanishing white matter disease’ (VWM), is a leukoencephalopathy with autosomal recessive inheritance. It is characterised by normal psychomotor development initially, with an onset of neurological deterioration that follows a chronic and progressive course. Stress conditions such as febrile infections, minor head trauma or even acute fright provoke major episodes of neurological deterioration. We present a case of a 2-year-old child who presented with spasticity and cerebellar ataxia. After magnetic resonance imaging (MRI) of the brain, CACH/VWM was diagnosed on the basis of the typical clinical and MRI findings. As there is no known cure for CACH/VWM, our patient was followed up over 3 years with MRIs of the brain to assess the progressive involvement of the cerebral white matter. In those patients with suggestive or inconclusive MRI findings for CACH/VWM, particularly in the presymptomatic stage and adult onset variants, involvement of the inner rim of the corpus callosum should prompt the inclusion of CACH/VWM in the differential diagnosis. Biochemical markers such as the asialotransferrin:transferrin ratio in the cerebrospinal fluid can also potentially be used as a screening tool in this subset of patients prior to gene mutation analysis.

## Introduction

Childhood ataxia and central nervous system hypomyelination (CACH), also known as ‘vanishing white matter disease’ (VWM) and ‘myelinopathia centralis diffusa’, is a leukoencephalopathy with autosomal recessive inheritance.^1,2,3^

A study in Canada in 1988 described severe infantile leukodystrophy amongst the native Cree Indian villages, termed ‘Cree leukoencephelophathy’,^4^ a condition that is now recognised as a phenotypic variant of CACH/VWM.^5^ Although VWM was initially described in young children, it is now well known that it has a wide phenotypic spectrum, affecting people of all ages, with the age of onset inversely proportional to the clinical severity.^1,2^ Childhood ataxia and central nervous system hypomyelination (vanishing white matter disease) is characterised by normal psychomotor development initially, with an onset of neurological deterioration that follows a chronic and progressive course.^1,2,3^ Stress conditions such as febrile infections, minor head trauma or even acute fright provoke major episodes of neurological deterioration in this condition.^12,3^

The exact prevalence of VWM is unknown; however, it may be one of the more common leukodystrophies.^3^ In a study of unclassified leukodystrophies in childhood by Van der Knaap et al., CACH was the most common, with 21 of a total of 92 patients found to have magnetic resonance imaging (MRI) features of CACH/VWM.^6^ This article aims to provide more insight into CACH/VWM, with a focus on the progression of the MRI brain imaging findings in our patient, who was imaged over 3 years. Our understanding of CACH/VWM continues to evolve, with increasing literature over the last two decades providing a better understanding of the imaging findings and genetic mutations.

## Case report

We present a case of a 2-year-old child who initially presented with cerebellar ataxia and spasticity and was subsequently booked for MRI of the brain. The provisional diagnosis was that of CACH/VWM based on the clinical presentation and classical MRI findings.

## Magnetic resonance imaging (findings over 3 years)

**FIGURE 1:**
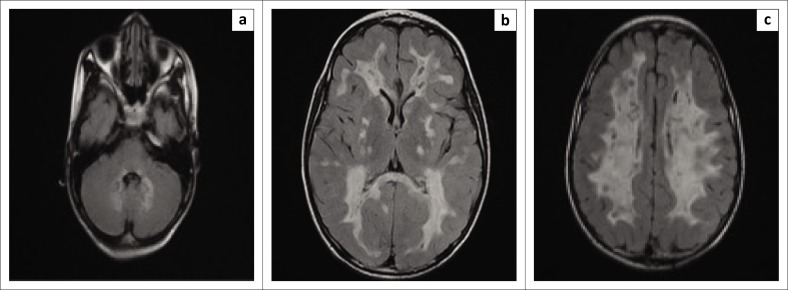
Initial magnetic resonance imaging (MRI) (after clinical presentation): (a) axial fluid attenuation inversion recovery (FLAIR) image with high signal in the cerebellar white matter tracts with associated mild cystic degeneration. (b and c) Axial FLAIR images show a classical MRI picture of CACH/VWM – diffuse symmetric signal abnormality with mild cystic degeneration in the cerebral white matter, sparing the U fibres. CACH/VWM, childhood ataxia and central nervous system hypomyelination (vanishing white matter disease).

The initial MRI (Figure 1a–c) demonstrated diffuse, symmetrical, white matter signal abnormalities with mild cystic degeneration and involved the corpus callosum, basal ganglia and cerebellar white matter. These regions corresponded with high signal intensity on the T2 weighted image (T2WI), proton density (PD) and fluid attenuation inversion recovery (FLAIR) sequences and showed no enhancement post-gadolinium. There was sparing of the subcortical white matter.

**FIGURE 2:**
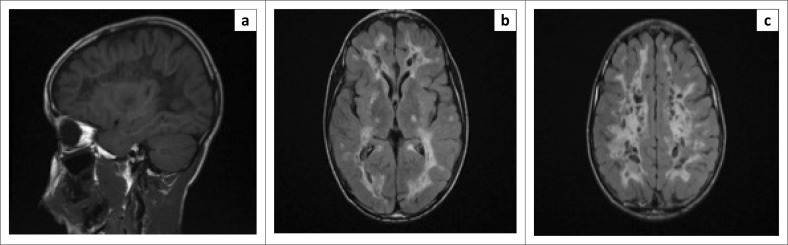
Magnetic resonance imaging after 1 year: (a) parasagittal T1 weighted imaging (T1WI) demonstrates radiating stripes stretching across the rarefied white matter, which is suggestive of the remaining normal white matter tracts. (b and c) Axial fluid attenuation inversion recovery (FLAIR) images with significant progression in the white matter signal abnormality and cystic degeneration.

**FIGURE 3:**
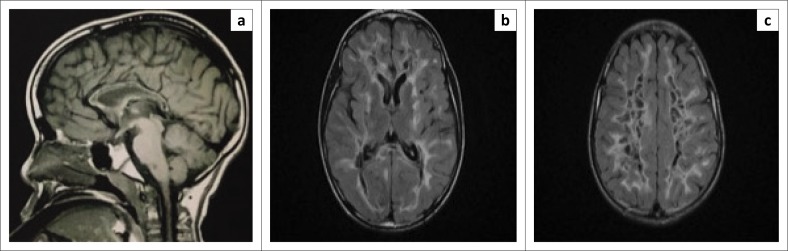
Magnetic resonance imaging after 2 years: (a) sagittal T1 weighted imaging (T1WI) demonstrates involvement of the inner rim of the corpus callosum. (b and c) Axial fluid attenuation inversion recovery (FLAIR) images with further progressive signal abnormalities and cystic degeneration of the white matter.

A radiating linear pattern within the abnormal white matter was suggestive of the remaining normal white matter tracts (Figure 2a). The corpus callosum involvement was limited to the inner rim and spared the outer rim (Figure 3a). Diffusion-weighted images showed an increase in diffusivity of the abnormal white matter, which was likely secondary to the rarefaction and cystic degeneration.

Follow-up MRI of the brain after 1 and 2 years (Figure 2a–c and Figure 3a–c) demonstrated progressive cystic degeneration in a radial pattern of the affected deep white matter over time, with signal intensity changing to that of cerebrospinal fluid (CSF) on T2WI, FLAIR and PD sequences.

## Discussion

Childhood ataxia and central nervous system hypomyelination (vanishing white matter disease) is caused by mutations in any of the five genes encoding the five subunits of the eukaryotic translational initiation factor 2B (eIF2B).^2^ Although eIF2B mutations have previously been described with an affinity for cerebral white matter only, it is now known that there is an association with ovarian failure.^7^ Gynaecological studies have demonstrated that the gene for VWM is located on chromosome 3q27.^8^ Prior to the availability of genetic testing for CACH/VWM, the diagnosis of this entity was based on typical clinical and MRI findings.^2^

The proposed diagnostic criteria for VWM include the following:^9^

- Initial motor and mental development is normal or near normal.
- Neurological deterioration has an early-childhood onset with an episodic and chronic progressive course.
- Neurological signs of spasticity and cerebellar ataxia (epilepsy and optic atrophy may also occur, but are not mandatory).
- Magnetic resonance imaging findings of symmetrical and bilateral involvement of the cerebral white matter, with parts or all of the white matter demonstrating signal intensities similar to that of CSF over time.

Childhood ataxia and central nervous system hypomyelination (vanishing white matter disease) can be diagnosed on the account of all four obligatory criteria being met.^9^

Van der Knaap et al. have described obligatory and suggestive MRI criteria for the diagnosis of VWM in those patients with classical MRI findings (Table 1); however, the use of these criteria is not applicable to identify the rare and atypical MRI variants.^10^

**TABLE 1:** Magnetic resonance imaging criteria for the diagnosis of vanishing white matter.

Obligatory	Suggestive
Cerebral white matter shows either diffuse or extensive signal abnormalities; the immediately subcortical white matter may be spared.	Within abnormal white matter, there is a pattern of radiating stripes on sagittal and coronal T1-weighted or FLAIR images; on axial images, dots and stripes are seen within the abnormal white matter as cross-sections of the stripes.
Part or all of the abnormal white matter has a signal intensity close to or the same as cerebrospinal fluid on proton density or FLAIR images, suggestive of white matter rarefaction or cystic destruction.	Lesions are seen within the central tegmental tracts in the pontine tegmentum.
If proton density and FLAIR images suggest that all cerebral white matter has disappeared, there is a fluid-filled distance between ependymal lining and the cortex, but not a total collapse of the white matter.	The corpus callosum involvement was limited to the inner rim and spared the outer rim.
The disappearance of the cerebral white matter occurs in a diffuse ‘melting away’ pattern.	-
The temporal lobes are relatively spared, in the extent of the abnormal signal, degree of cystic destruction or both.	-
Cerebellar white matter may be abnormal, but does not contain cysts.	-
There is no contrast enhancement.	-

*Source*: Van der Knaap MS, Pronk JC, Scheper GC. Vanishing white matter disease. Lancet Neurol. 2006;55:413–423. https://doi.org/10.1016/S1474-4422(06)70440-9

FLAIR, fluid attenuation inversion recovery.

All patients with CACH/VWM demonstrate MRI abnormalities, even the symptomless at-risk siblings of a proband.^3^ Magnetic resonance imaging typically shows diffuse and symmetrical involvement of the cerebral white matter with sparing of the U fibres, internal capsules and outer part of the corpus callosum.^1^ The cortical grey matter is always spared.^1^ Over time there is progressive rarefaction and cystic degeneration of the white matter that is eventually totally replaced by CSF-like signal intensity on T1WI, T2WI and FLAIR images (Figure 1a–c, Figure 2a–c, Figure 3a–c).^11^

On T1WI and FLAIR images, radiating stripes may be seen stretching across the rarefied white matter, which are suggestive of the remaining normal white matter tracts (Figure 2a).^3^ The cerebellar white matter may be affected, as evidenced in our case (Figure 1a), and the brainstem may also demonstrate abnormal signal intensity, particularly in the pontine central tegmental tracts.^1^ Cerebellar atrophy may occur in late stages and ranges from mild to severe, primarily involving the vermis.^3^ Contrast enhancement does not occur in CACH/VWM and diffusion-weighted imaging shows increased diffusion in areas of rarefaction and cystic degeneration.^1,12^

In a study conducted by van der Lei et al. that looked at the characteristics of the early MRI findings in VWM, the MRI abnormalities were not always widespread and diffuse in the presymptomatic or early stages of VWM, in contrast to what was previously thought.^2^ The initial changes were noted in the periventricular and bordering deep white matter. The inner rim of the corpus callosum was involved in all patients in this study and was also evidenced in our case (Figure 3a).^2^ The follow-up of MRI imaging of these patients demonstrated the more classical diffuse, symmetrical MRI picture of VWM.^2^

Magnetic resonance spectroscopy (MRS) of the affected cerebral white matter demonstrates a stage-dependant reduction of the normal metabolite peaks with the accumulation of glucose and lactate at CSF-like concentrations.^1,10^ Loss of the normal N-acetyl aspartate, choline and creatine peaks are a result of the rarefaction and cystic degeneration.^13^ The MRS findings at the end stage of this spectrum are not diagnostic for CACH/VWM, as they may be evident in any cystic white matter disease.^10^

Cerebrospinal fluid analysis may demonstrate a decreased asialotransferrin:transferrin ratio, which has a high sensitivity and specificity for patients with eIFB2-related mutations.^14^ This biochemical marker offers the potential to be used as a screening tool in selected cases prior to expensive DNA sequencing tests in patients with suggestive or inconclusive MRI findings.^14^

In individuals with a rapid neurological decline following a febrile infection, the differential for CACH/VWM includes acute disseminated encephalomyelitis (ADEM) and encephalitis.^2^ Unlike CACH/VWM, children with ADEM may have evidence of inflammation in the CSF with MRI of the brain typically showing asymmetric, multifocal, white matter abnormalities. Encephalitis demonstrates variable lesions in the white, as well as grey matter, where CACH/VWM only involves the white matter.^2^ In both ADEM and encephalitis, contrast enhancement may be apparent, which is not evident in CACH/VWM.^2^

The differential diagnosis for CACH/VWM also includes other inherited disorders presenting in infancy and childhood with progressive neurological deterioration and diffuse white matter abnormalities. Alexander disease demonstrates increasing frontoparietal white matter atrophy with cystic degeneration and, unlike CACH/VWM, contrast enhancement of selected grey and white matter structures are characteristic of this disease.^3^

Macrocephaly is present in patients with Alexander disease but may also occur in selected cases of CACH/VWM.^15^ In X-linked adrenoleukodystrophy, metachromatic leukodystrophy, Krabbe disease and Canavan disease, diffuse cerebral white matter changes occur but in the absence of cystic degeneration.^3^ In megalencephalic leukoencephalopathy with subcortical cysts, MRI shows diffuse supratentorial white matter abnormalities, subcortical cysts mostly in the frontoparietal border zone region and anterior-temporal subcortical white matter and, unlike CACH/VWM, the cerebral white matter does not undergo diffuse rarefaction and cystic degeneration.^3^ The imaging appearance of mitochondrial disorders may appear similar to that of CACH/VWM with diffuse white matter rarefaction and cystic degeneration.^3^

## Conclusion

Childhood ataxia and central nervous system hypomyelination (vanishing white matter disease) is a chronic and progressive white matter disorder, often exacerbated by infection or minor head trauma. The proposed diagnostic and MRI criteria assist in making the diagnosis of CACH/VWM with a high degree of certainty in those patients with typical clinical and MRI findings. However, in those patients with suggestive or inconclusive MRI findings, involvement of the inner rim of the corpus callosum should prompt the inclusion of VWM in the differential diagnosis. Cerebrospinal fluid analysis of asialotransferrin:transferrin ratio can also be used in this subset of patients as a screening tool prior to gene testing.
